# RNA-seq Transcriptional Profiling of Peripheral Blood Leukocytes from Cattle Infected with *Mycobacterium bovis*

**DOI:** 10.3389/fimmu.2014.00396

**Published:** 2014-08-26

**Authors:** Kirsten E. McLoughlin, Nicolas C. Nalpas, Kévin Rue-Albrecht, John A. Browne, David A. Magee, Kate E. Killick, Stephen D. E. Park, Karsten Hokamp, Kieran G. Meade, Cliona O’Farrelly, Eamonn Gormley, Stephen V. Gordon, David E. MacHugh

**Affiliations:** ^1^Animal Genomics Laboratory, UCD School of Agriculture and Food Science, University College Dublin, Dublin, Ireland; ^2^Smurfit Institute of Genetics, Trinity College Dublin, Dublin, Ireland; ^3^Animal and Bioscience Research Department, Animal and Grassland Research and Innovation Centre, Dunsany, Ireland; ^4^Comparative Immunology Group, School of Biochemistry and Immunology, Trinity Biosciences Institute, Trinity College Dublin, Dublin, Ireland; ^5^Tuberculosis Diagnostics and Immunology Research Centre, UCD School of Veterinary Medicine, University College Dublin, Dublin, Ireland; ^6^UCD School of Veterinary Medicine, University College Dublin, Dublin, Ireland; ^7^UCD Conway Institute of Biomolecular and Biomedical Research, University College Dublin, Dublin, Ireland

**Keywords:** *Mycobacterium bovis*, tuberculosis, RNA-seq, biomarker, cattle, microarray, peripheral blood

## Abstract

Bovine tuberculosis, caused by infection with *Mycobacterium bovis*, is a major endemic disease affecting cattle populations worldwide, despite the implementation of stringent surveillance and control programs in many countries. The development of high-throughput functional genomics technologies, including gene expression microarrays and RNA-sequencing (RNA-seq), has enabled detailed analysis of the host transcriptome to *M. bovis* infection, particularly at the macrophage and peripheral blood level. In the present study, we have analyzed the peripheral blood leukocyte (PBL) transcriptome of eight natural *M. bovis*-infected and eight age- and sex-matched non-infected control Holstein-Friesian animals using RNA-seq. In addition, we compared gene expression profiles generated using RNA-seq with those previously generated using the high-density Affymetrix^®^ GeneChip^®^ Bovine Genome Array platform from the same PBL-extracted RNA. A total of 3,250 differentially expressed (DE) annotated genes were detected in the *M. bovis*-infected samples relative to the controls (adjusted *P*-value ≤0.05), with the number of genes displaying decreased relative expression (1,671) exceeding those with increased relative expression (1,579). Ingenuity^®^ Systems Pathway Analysis (IPA) of all DE genes revealed enrichment for genes with immune function. Notably, transcriptional suppression was observed among several of the top-ranking canonical pathways including *Leukocyte Extravasation Signaling*. Comparative platform analysis demonstrated that RNA-seq detected a larger number of annotated DE genes (3,250) relative to the microarray (1,398), of which 917 genes were common to both technologies and displayed the same direction of expression. Finally, we show that RNA-seq had an increased dynamic range compared to the microarray for estimating differential gene expression.

## Introduction

Bovine tuberculosis (BTB) is caused by infection with *Mycobacterium bovis*, a member of the *Mycobacterium tuberculosis* complex (MTC). The mycobacterial species and strains that constitute the MTC can cause tuberculosis in a wide range of mammals and display 99.9% similarity at the nucleotide level (1). Econometric analyses incorporating agricultural production and human health indices have placed BTB as the fourth most important disease of cattle, costing an estimated $3 billion on a global scale annually (2). Furthermore, as a zoonotic agent, *M. bovis* infection has important implications for human health (3, 4).

*Mycobacterium bovis* is normally transmitted via the inhalation of infectious bacilli, whereby infection is established in the lung. Evasion of host immune defenses enables the pathogen to survive and replicate within phagocytic macrophages, the primary innate immune cell that mediates the response to infection, and can result in dissemination of infection via the lymph system leading to disease progression and pathology (5). Subsequent transmission of the pathogen to susceptible hosts maintains the cycle of infection. The immune response to BTB is complex and is largely characterized by macrophage-mediated development of protective T_H_1-type responses following initial exposure to the pathogen. It has been reported that the development of disease involves a transition from T_H_1 to non-protective T_H_2-type responses (6). The progression of infection may also be due to the modulation and suppression of specific immune mechanisms by the pathogen (5).

In many developed countries, control and eradication programs have been put in place to facilitate early detection and removal of infected animals. In Ireland, the test and slaughter policy was introduced in the early 1950s as part of the national BTB eradication scheme (7). This policy includes mandatory screening of animals in the national herd using the single intradermal comparative tuberculin test (SICTT), alone or in combination with *in vitro* ELISA-based interferon-gamma (IFN-γ) release assays in infected herds, to increase the sensitivity of diagnosis (8). However, due to limitations in both of these tests and also the presence of wildlife reservoirs (including the Eurasian badger, *Meles meles*) *M. bovis* has remained recalcitrant to eradication (6). Consequently, there is a pressing need to develop novel diagnostics for early and reliable detection of *M. bovis* infection in cattle herds.

The availability of a well-annotated bovine genome sequence with concomitant advances in high-throughput genomics technologies offers novel approaches to interrogate and better understand the immune response to *M. bovis* infection. Many transcriptomics studies of the host response to *M. bovis* have involved the analysis of blood-derived RNA from naturally or experimentally infected animals, as previous work has shown that for BTB, host immune responses occurring in peripheral blood reflect those at the primary site of disease (9). Microarray analysis of peripheral blood-derived RNA has shown that transcriptional profiling can unambiguously differentiate animals by disease status and can identify immunomodulatory mechanisms associated with pathology (10–13).

The advent of high-throughput sequencing technologies has given rise to new methods for gene expression analysis based on RNA-sequencing (RNA-seq). This RNA-seq approach has a number of important advantages compared to microarray analysis, including unbiased whole-transcriptome profiling; the characterization and analysis of both sense and antisense transcription and novel transcripts; the identification of mRNA isoforms; increased precision and sensitivity for the quantification of lowly expressed transcripts; and the detection of expressed coding and regulatory DNA sequence variants that can influence phenotype [e.g., disease resistance and susceptibility] (14–16).

To gain a deeper knowledge of the host transcriptional response to *M. bovis* infection, we have used RNA-seq to compare the peripheral blood leukocyte (PBL) transcriptomes of eight animals naturally infected with *M. bovis* and eight non-infected control animals. Differentially expressed (DE) genes identified from this analysis were further investigated using the Ingenuity^®^ Systems Pathway Analysis (IPA) Knowledgebase to detect overrepresented cellular pathways in response to *M. bovis* infection. We also compared the gene expression profiles generated from RNA-seq with data from the Affymetrix^®^ GeneChip^®^ Bovine Genome Array using the same PBL-extracted RNA samples (12).

## Materials and Methods

### Animals

The 16 age-matched female Holstein-Friesian animals used in this study have been previously described (12). The eight *M. bovis*-infected cattle were selected from a panel of naturally infected animals identified during routine disease surveillance by the Irish Department of Agriculture, Food and the Marine. These animals had a positive result for both SICTT and whole blood IFN-γ-based BOVIGAM^®^ assay tests (Prionics AG, Zurich, Switzerland). In addition, *M. bovis* infection was confirmed following detailed post-mortem pathological examination and culture. Non-infected control animals were selected from a herd with no recent history of *M. bovis* infection and were shown to be negative for both the SICTT and IFN-γ tests. All animal procedures detailed were performed according to the provisions of the Cruelty to Animals Act (licenses issued by the Irish Department of Health and Children) and ethics approval for the study was obtained from the UCD Animal Ethics Committee.

### Blood collection and RNA extraction

The materials and methods used to isolate and purify PBL-derived RNA from all 16 animals have been described by us previously. Briefly, whole blood was collected from each animal in 8 ml heparin vacutainers^®^ (Becton-Dickinson Ltd., Dublin, Ireland) and RNA extraction was performed within 2 h of blood collection. The complete methods used for blood collection, PBL isolation, and total RNA extraction and purification have been described by us previously (12). RNA quantity and quality checking was performed using the NanoDrop™ 1000 spectrophotometer (Thermo Fisher Scientific, Waltham, MA, USA) and the Agilent 2100 Bioanalyzer using an RNA 6000 Nano LabChip kit (Agilent Technologies, Cork, Ireland). All samples displayed a 260/280 ratio >1.8 and RNA integrity numbers (RIN) >8.0.

### RNA-seq library preparation

The laboratory method used to generate RNA-seq libraries was adapted from a protocol previously described by our group (17). In total, 16 strand-specific RNA-seq Illumina^®^ libraries were prepared (i.e., eight libraries each for the infected and control groups) using 1.2 μg of total RNA. Total RNA was heated at 65°C for 5 min to disrupt any secondary structure and purification of poly(A) RNA was performed using a Dynabeads^®^ mRNA DIRECT^®^ Micro Kit according to the manufacturer’s instructions (Invitrogen™). Purified poly(A) RNA was then fragmented using 1× RNA Fragmentation Reagent (Ambion^®^/Life Technologies Ltd., Warrington, UK) for 5 min at 70°C and precipitated using 68 mM sodium acetate pH 5.2 (Ambion^®^), 227 ng/μl glycogen (Ambion^®^), and 30 μl of 100% ethanol (Sigma-Aldrich Ltd., Dublin, Ireland). Pellets were washed with 80% ethanol, air-dried for 10 min at room temperature, and re-suspended in 10.5 μl DNase- and RNase-free water.

Synthesis of first strand cDNA was performed by incubating fragmented RNA with 261 mM Random Hexamer Primers (Invitrogen™), 1× first strand buffer (Invitrogen™); 10 mM DTT (Invitrogen™); 0.5 mM dNTPs; 20 U RNaseOUT™ recombinant ribonuclease inhibitor; and 200 U SuperScript^®^ II Reverse Transcriptase (Invitrogen™) at 25°C for 10 min, at 42°C for 50 min, and 70°C for 15 min. First strand synthesis reaction mixtures were purified using MicroSpin G-50 columns according to the manufacturer’s instructions (GE Healthcare UK Ltd., Buckinghamshire, UK).

Second strand cDNA synthesis, involving the incorporation of uracil, was performed by adding the first strand cDNA synthesis reaction to a second strand reaction mix consisting of 0.065× first strand buffer (Invitrogen™); 1× second strand buffer (Invitrogen™); a dNTP mix consisting of a final concentration of 0.3 mM dATP, dCTP, dGTP (Sigma-Aldrich) and 0.3 mM dUTP (Bioline Reagents Ltd., London, UK); 1 mM DTT (Invitrogen™); 2 U RNase H (Invitrogen™) and 50 U *E. coli* DNA Polymerase I (Invitrogen™). Reactions were incubated at 16°C for 2.5 h. The double stranded cDNA was subsequently purified using a QIAquick PCR Purification kit (Qiagen) according to the manufacturer’s instructions and eluted in 30 μl of the provided elution buffer.

Blunt-end repair of cDNA was performed in a 100 μl reaction containing 1× T4 DNA ligase buffer with 10 mM dATP (New England Biolabs^®^ Inc., Ipswich, MA, USA), 0.4 mM of each dNTP (Invitrogen™), 15 U T4 DNA polymerase (New England Biolabs^®^), 5 U DNA Polymerase I Large [Klenow] Fragment (New England Biolabs^®^), and 50 U T4 polynucleotide kinase (New England Biolabs^®^). Reactions were incubated at 20°C for 30 min and the cDNA was then purified using a QIAquick PCR Purification Kit (Qiagen) according to the manufacturer’s instructions and eluted in 32 μl of the provided elution buffer.

To facilitate Illumina^®^ GA adaptor ligation, a single “A” base was added to the 3′ ends of the blunt-end-repaired cDNA samples. Thirty-two microliters of purified phosphorylated blunt-end-repaired cDNA was included in a final 50 μl reaction mixture containing 1× Klenow fragment buffer (New England Biolabs^®^); 0.2 mM dATP (Invitrogen™), and 15 U Klenow fragment with 3′-to-5′ exonuclease activity (New England Biolabs^®^). Reactions were incubated at 37°C for 30 min, after which cDNA was purified using a QIAquick MinElute Kit (Qiagen) according to the manufacturer’s instructions and eluted in 21 μl of the provided elution buffer.

Illumina^®^ RNA-seq adaptor ligation reactions (50 μl volumes) involved incubation of 21 μl of phosphorylated blunt-ended cDNA containing a 3′-dATP overhang with 1× Quick DNA ligase buffer (New England Biolabs^®^); 30 nM custom indexed single-read adaptors (see Table S1 in Supplementary Material for barcode index sequences); and 15 U T4 DNA ligase (Invitrogen™). Reaction mixes were incubated at room temperature for 15 min and purified using a QIAquick MinElute Kit according to the manufacturer’s instructions (Qiagen) and eluted in 10 μl of the provided elution buffer. Adaptor-ligated cDNA was gel-purified using 2.5% agarose gels stained with 1× SYBR^®^ Safe DNA gel stain (Invitrogen™). Gels were electrophoresed at 100 V using 1× TAE buffer (Invitrogen™) for 75 min at room temperature. Size fractionated bands corresponding to 200 bp (+50 bp) were excised from each sample and purified using a QIAquick Gel Extraction kit (Qiagen) according to the manufacturer’s instructions and eluted in 30 μl of elution buffer. To generate strand-specific RNA-seq libraries, the second strand of the gel-purified adapter-ligated cDNA containing uracil was digested enzymatically in 30 μl reaction volumes containing 1× Uracil-DNA Glycosylase buffer and 1 U Uracil-DNA Glycosylase (Bioline). Reactions were incubated at 37°C for 15 min followed by 94°C for 10 min.

PCR enrichment amplifications (25 μl) were performed and contained 9 μl of second strand-digested, adaptor-ligated cDNA; 1× Phusion^®^ High-Fidelity DNA polymerase buffer (New England Biolabs^®^); 334 nM each Illumina^®^ PCR primer (Illumina^®^ Inc., San Diego, CA, USA); 0.4 mM each of dATP, dCTP, DGTP, and dTTP (Invitrogen™) and 1 U Phusion^®^ High-Fidelity DNA polymerase (New England Biolabs^®^). PCR amplification reactions consisted of an initial denaturation step of 98°C for 30 s, 18 cycles of 98°C for 10 s, 65°C for 30 s, and 72°C for 30 s, followed by a final extension step of 72°C for 5 min. PCR products were visualized following electrophoresis on a 2% agarose gel stained with 0.25× SYBR^®^ Safe DNA gel stain (Invitrogen™) and purified to remove PCR-generated adaptor-dimer using an Agencourt AMPure XP kit (Beckman Coulter Genomics, Danvers, MA, USA) according to the manufacturer’s instructions with final elution in 30 μl of 1× TE buffer.

All RNA-seq libraries were quantified using a Qubit^®^ Fluorometer (Invitrogen™). RNA-seq library quality was assessed using an Agilent Bioanalyzer and Agilent High sensitivity DNA chip (Agilent) and confirmed that library insert sizes were ~200–250 bp for all individual libraries. Individual RNA-seq libraries were standardized and pooled in equimolar quantities (10 μM for each individual library). The quantity and quality of the final pooled library was assessed as described above prior to sequencing.

The libraries were subsequently validated using conventional Sanger sequencing of individual library clones. Library fragments from two libraries were cloned using the Zero Blunt^®^ TOPO^®^ PCR Cloning system according to the manufacturer’s instructions (Invitrogen™). Conventional Sanger sequencing of 10 plasmid inserts from each of the 2 libraries confirmed that the RNA-seq libraries contained inserts derived from bovine mRNA. Plasmid sequencing was outsourced (Source Bioscience Ltd., Dublin, Ireland) and sequences generated were validated using BLAST-searching of the DNA sequence database (18).

Cluster generation and sequencing of the pooled RNA-seq library was performed on an Illumina^®^ Cluster Station and Genome Analyzer IIx sequencer according to the manufacturer’s instructions. The pooled library was sequenced as single-end read 84-mers using Illumina^®^ version 4.0 sequencing kits and the standard Illumina^®^ Genome Analyzer IIx pipeline. The Illumina^®^ Sequencing Control Software version 2.9 and Real-Time Analysis version 1.9 software packages were used for real-time tracking of the sequencing run, real-time image processing, the generation of base intensity values, and base calling. These RNA-seq data have been deposited in the NCBI Gene Expression Omnibus (GEO) database with experiment series accession number GSE60265.

### Bioinformatics and statistical analysis of RNA-seq and microarray data

All the bioinformatics pipeline bash, Perl, and R scripts used for computational analyses were deposited in a GitHub repository at https://github.com/kmcloughlin1 and these analyses were performed on a 32-node Compute Server running Linux Ubuntu (version 12.04.2).

An initial quality check was performed on each of the raw read data files using the FastQC software (version 0.10.1)^1^ to determine the best sequence read quality filtering strategy. Subsequently, a custom perl script was used to: (1), deconvolute the pooled libraries into individual libraries of sample sequence reads based on the unique index barcode (allowing up to one mismatch as long as the barcode sequence can be associated to a single unique index barcode); (2) filter out single-end reads containing adaptor sequence (allowing up to three mismatches); and (3) remove single-end reads of poor quality (i.e., reads containing 25% of bases with a Phred quality score below 20). Filtered individual libraries file were checked again with the FastQC software package to confirm sequence read quality. Single-end reads, from each filtered individual sample library, were aligned to the *Bos taurus* reference genome [UMD3.1.73; (19)] using the STAR aligner software package [version 2.3.0] (20).

For each library, raw counts for each annotated gene were obtained using the featureCounts software from the Subread package [version 1.3.5-p4] (21). The featureCounts parameters were set to unambiguously assign uniquely aligned single-end reads in a stranded manner to the exons of genes within the *B. taurus* reference genome annotation (UMD3.1.73 genome annotation).

Differential gene expression analysis was performed using the gene raw counts, obtained from featureCounts, within the Bioconductor edgeR package (22). The differential gene expression pipeline within the edgeR package was customized to (1) filter out all bovine rRNA genes; (2) filter out genes displaying expression levels below the minimally set threshold of one count per million (CPM) in at least eight individual libraries (i.e., equivalent to one group of biological replicates); (3) calculate normalization factors for each library using the trimmed mean of *M*-values method (14); (4) generate the density of counts per gene and multidimensional scaling (MDS) plots based on data from each individually barcoded library (using the Euclidean distance metric); (5) estimate the dispersion parameter for each library using the Cox-Reid method; (6) identify DE genes between infected versus non-infected control samples (i.e., unpaired-sample statistical model) using a negative binomial generalized linear model; and (7) adjust the *P*-value for multiple testing using the Benjamini–Hochberg correction (23) with a false discovery rate (FDR) ≤0.05. Mean fold-changes in gene expression are reported in the main body text as geometric mean values in the *M. bovis*-infected group relative to the control group; for genes displaying reduced relative expression, the negative reciprocal geometric mean fold-changes are given.

The IPA^®^ Knowledgebase^2^ was used to identify cellular pathways and gene ontology categories that were overrepresented based on the list of DE genes (*P-*value ≤0.05).

The raw microarray data generated from the same 16 PBL-extracted RNA samples were retrieved from the NCBI GEO repository (24) with the accession number GSE33359 (12). The Affymetrix^®^ GeneChip^®^ Bovine Genome Array used to generate these data contains 24,072 probe sets representing more than 23,000 gene transcripts. To compare gene expression profiles from these samples using RNA-seq and the microarray, we first re-analyzed the microarray data using a series of Bioconductor packages (25) and the most recent build of the bovine genome [UMD3.1.73; (19)]. Normalization of raw data was performed using the Factor Analysis for Robust Microarray Summarization (FARMS) algorithm (26). The FARMS algorithm uses only perfect match (PM) probes and a quantile normalization procedure, providing both *P*-values and signal intensities. Normalized data were then further subjected to filtering for informative probes sets using the informative/non-informative (I/NI) calls unsupervised feature selection criterion implemented in FARMS (27). This defines a probe set as being informative when many of its probes reflect the same change in mRNA concentration across arrays. To compare and contrast the two gene expression technologies, microarray probe sets were first annotated with the corresponding Ensembl ID using the Bioconductor biomaRt package (28). Genes displaying differential expression between control and infected groups were identified using the linear models for microarray data (LIMMA) bioconductor package (29). Following this, a Benjamini–Hochberg multiple-testing correction of ≤0.05 was applied to all DE genes (23) and the Euclidean distance was used as the distance metric for MDS plotting.

## Results

### Summary statistics for the RNA-seq data

All 16 RNA-seq libraries were sequenced across one full Illumina^®^ GAIIX flow cell. Deconvolution and filtering of sequence reads to remove adaptor-dimer contamination yielded a mean of 13.2 million reads per individual barcoded RNA-seq sample library. Alignment of the filtered reads to the *B. taurus* UMD3.1.73 genome build yielded a mean of 11.8 million reads (90%) that aligned to unique locations in the bovine genome for each RNA-seq library; a mean of 906,679 reads (7%) for each library that aligned to multiple locations in the genome; and a mean of 381,991 reads (3%) for each library that did not align to any genome location (Table S1 in Supplementary Material). Further analysis of the mean 11.8 million reads mapping to unique genome locations demonstrated that 52% of these mean reads were assigned to annotated regions of the genome, which were used to calculate raw counts for each sense gene and subsequently used for downstream bioinformatics and systems analyses; while 48% were not assigned to any annotated genome location or were assigned to overlapping (therefore ambiguous) annotated genomic regions.

### Gene expression and IPA analysis of sense strand transcription

Analysis of the gene coverage based exclusively on sense strand sequence information, revealed that of the 24,616 annotated *B. taurus* genes in Ensembl (release 73), 17,792 genes (72.3%) had at least one sequence read count (i.e., one mapped read) in at least 1 of the 16 individual sample RNA-seq libraries. The 17,792 detectable genes were further filtered by removing lowly expressed genes, whereby only genes displaying more than 1 CPM reads in 8 or more individual libraries were used for subsequent analyses. This yielded 12,294 genes (49.9% of annotated *B. taurus* genes) that were suitable for downstream analyses.

**Figure 1:**
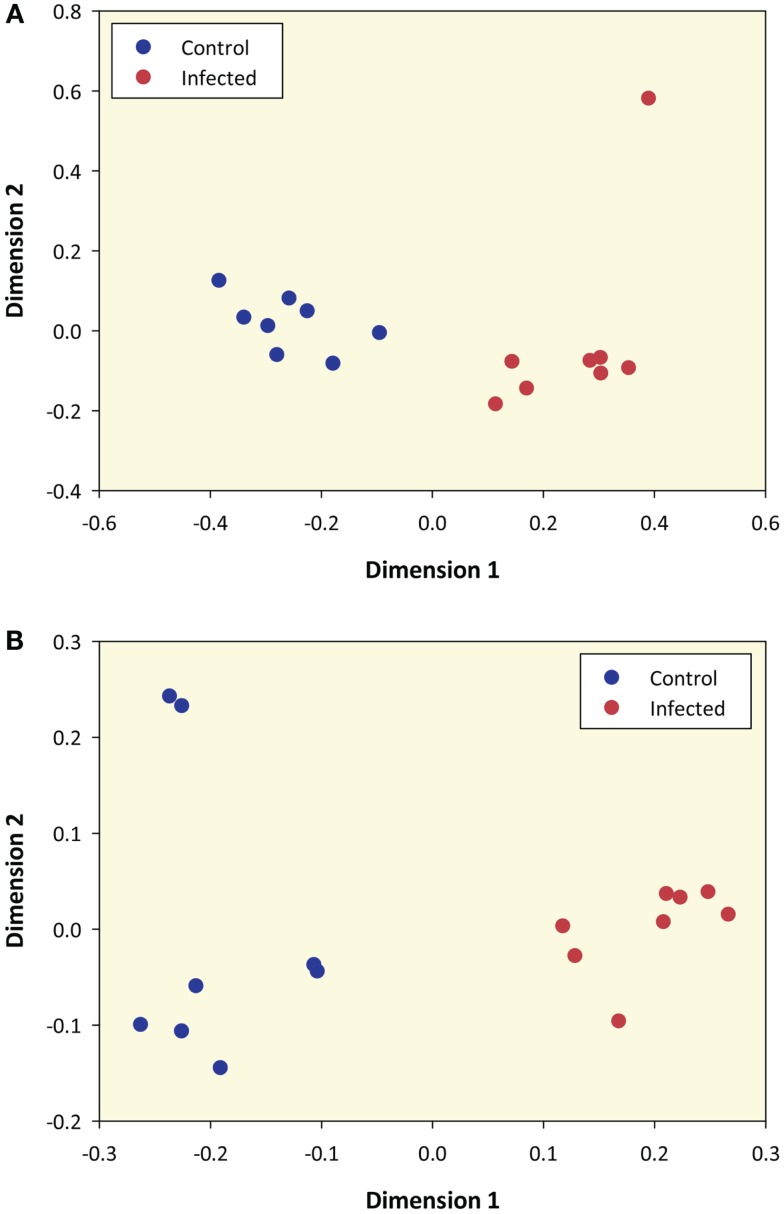
**Multidimensional scaling plot of *M. bovis*-infected and control samples based on RNA-seq sense data and microarray data**. **(A)** MDS plot of 8 *M. bovis*-infected and 8 control PBL samples generated from 12,294 genes that passed all data filtering prior to differential gene expression (based on RNA-seq sense data only). **(B)** MDS plot using data from 5,082 informative microarray probe sets in 8 *M. bovis*-infected and 8 control PBL samples.

Prior to differential gene expression analysis, the 12,294 filtered genes were used to generate an MDS plot to visualize gene expression and infection status for the 16 animal PBL samples (Figure 1A). This plot shows that samples were clearly differentiated according to infection status along dimension 1 and dimension 2 highlights one *M. bovis*-infected sample as a possible outlier (*Infected 34* – animal ID).

Statistical analysis of all 12,294 genes that passed the filtering process identified a total of 3,250 DE genes (FDR ≤0.05), of which 1,579 and 1,671 displayed increased and decreased expression, respectively, in the *M. bovis*-infected samples relative to the non-infected control samples (Table S2 in Supplementary Material). Among the DE genes showing the greatest mean fold-change increase in expression were *CXCL6* (+6.77), *IL8* (+5.39), and *CTLA4* (+3.61), while *CXCL10* (−3.27), *DEFB10* (−7.21), and *IL12* (−4.35) showed the greatest mean fold-change decrease in expression; all of these genes have been previously shown to be involved in the host response to mycobacterial infection (12, 30–33).

**Figure 2:**
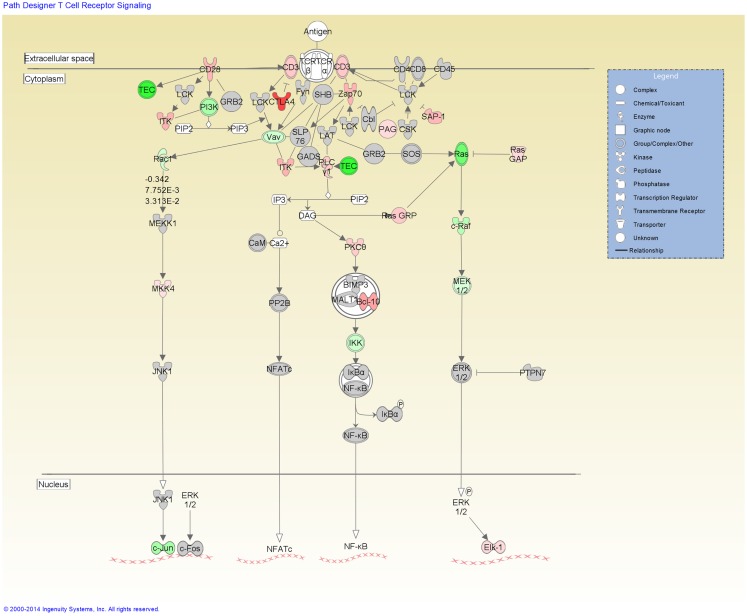
**The top-ranked enriched canonical pathway identified using IPA, the *T-cell receptor signaling* pathway**. Red shading indicates increased expression in *M. bovis*-infected animals relative to the non-infected control group. Green shading indicates decreased expression in *M. bovis*-infected animals relative to the non-infected control group.

**Figure 3:**
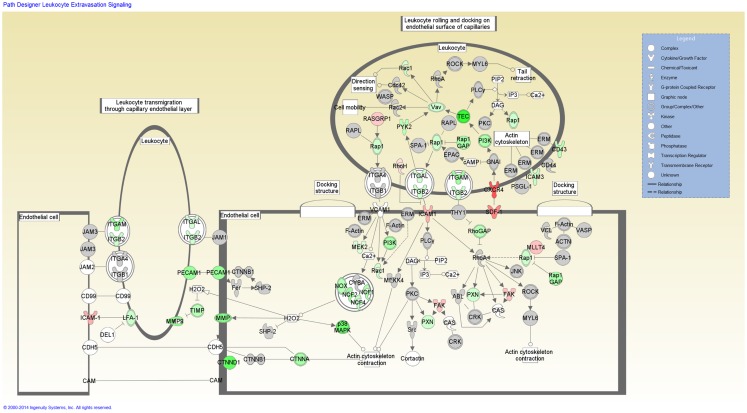
**The second-ranked enriched canonical pathway identified using IPA, the *Leukocyte extravasation* pathway**. Red shading indicates increased expression in *M. bovis*-infected animals relative to the non-infected control group. Green shading indicates decreased expression in *M. bovis*-infected animals relative to the non-infected control group.

Functional categorization of the DE genes using IPA revealed an overrepresentation of genes with roles in inflammatory response, immunological disease and infectious disease. Of the 3,250 genes found to be DE, 2,785 mapped to the IPA Knowledgebase. IPA analysis identified 201 statistically significant (*P-*value ≤0.05) enriched pathways, many of which were associated with immune function (Table S3 in Supplementary Material). Based on the well-documented role of the T-cell response to mycobacterial infection, the top-ranking IPA canonical pathway (*T-cell receptor signaling*) was overlaid with the RNA-seq gene expression data, which indicated activation of this pathway (Figure 2). Further inspection of the IPA results showed *Leukocyte extravasation signaling* to be the second-ranked canonical pathway. Transcriptional suppression was observed for this pathway with several genes required for migration of leukocytes to the site of infection displaying reduced relative expression (Figure 3). These include genes encoding leukocyte ligands required for endothelial adhesion such as *ITGB2* (−1.29-fold), *ITGAL* (−1.23-fold), and *SPN* (also known as *CD43*; −1.42-fold). Reduced relative expression of the gene encoding the LFA-1 protein was also indirectly observed in these BTB-infected animals. LFA-1 is a complex formed from an α-chain encoded by *ITGAL* and a β-chain encoded by *ITGB2*; both *ITGAL* and *ITGB2* exhibited decreased relative expression as discussed above. Decreased relative expression of *PECAM1* (−1.69-fold), which encodes a protein involved in the transmigration of leukocytes through or between endothelial layers into tissues during extravasation, was also detected in the present study (34). Furthermore, a reduction in relative expression was also observed for *MMP9* (−1.79), which degrades the extracellular matrix facilitating the transmigration of leukocytes (35, 36).

### Comparison of the number of differentially expressed genes identified from RNA-seq and microarray platforms

The RNA extracted from these animals has previously been analyzed using the Affymetrix^®^ Bovine Genome Array (12). To directly compare gene expression profiles generated by the two platforms, we re-analyzed all microarray data and of the 24,072 probe sets represented on the microarray, 5,082 of these passed the filtering process and were designated as informative. An MDS plot generated from these 5,082 informative probe sets shows samples clustered according to their infection status (Figure 1B); this pattern was also observed for these samples by Killick and colleagues using hierarchical clustering (12).

Analysis of all informative microarray probe sets identified 2,808 DE transcripts (FDR ≤0.05) and mapping of these transcripts to the *B. taurus* UMD3.1.73 genome build yielded 1,398 DE genes with Ensembl IDs (Table S4 in Supplementary Material). This is substantially lower than the 2,757 DE genes previously reported by us for the same RNA samples (12). This discrepancy may be explained by differences in the versions of the *B. taurus* reference genome used to annotate the microarray probe sets: for the previous study, we used the Btau4.0 genome assembly, while in the current study the UMD3.1.73 genome assembly was used. Discrepancies between the two genome annotations as detailed by Zimin et al. (37) most readily account for the reduced number of DE genes with Ensembl IDs observed for the present study.

**Figure 4:**
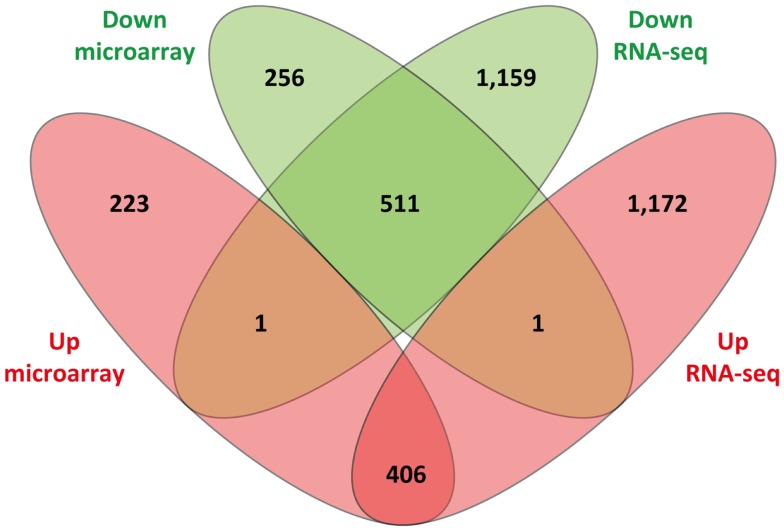
**Venn diagram showing comparison of differentially expressed genes identified from sense RNA-seq data and the microarray**. Sets of upregulated genes are represented in red and sets of downregulated genes are in green.

Of the 1,398 DE genes obtained from re-analysis of the Affymetrix^®^ Bovine Genome Array in infected animals relative to the control animals, 630 and 768 exhibited increased and decreased expression, respectively. Consequently, it is noteworthy that the number of Ensembl-annotated DE genes obtained with the RNA-seq analysis (3,250 DE genes; 1,579 increased relative expression and 1,671 decreased relative expression) was markedly higher than the number of DE genes detected using the microarray. Further examination of the results showed that 917 DE Ensembl genes had the same direction of expression (i.e., increased or decreased relative expression) on both platforms; 2,331 DE Ensembl genes were unique to the RNA-seq results (i.e., DE using RNA-seq but not DE on the microarray platform); and 479 DE Ensembl genes were unique to the microarray (i.e., DE on the microarray platform but not DE on the RNA-seq platform). Finally, two of the genes were found to have conflicting patterns of expression, i.e., genes that displayed increased relative expression on one platform and decreased relative expression on the other platform. *CHTOP* showed decreased relative expression on the RNA-seq platform but increased relative expression on the microarray platform and *GPR89* showed increased relative expression on the RNA-seq platform but decreased relative expression on the microarray platform (Figure 4).

Finally, analysis and comparison of the number of IPA-identified statistically significant canonical pathways (*P-*value ≤0.05) for the DE genes from both platforms revealed 101 pathways that were common to both, 100 that were unique to RNA-seq, and 36 that were unique to the microarray. The larger number of IPA-identified canonical pathways from the RNA-seq results reflects the greater number of DE genes detected using this platform. Despite this, however, there was a notable level of overlap in the number of pathways identified using both platforms. The percentage of overlapping pathways between the two platforms was estimated as 50.2% for RNA-seq and 73.7% for the microarray (Tables S3 and S5 in Supplementary Material).

### Correlation of the log fold-change in gene expression between the two platforms using all genes that passed filtering

We analyzed the correlation between the log_2_ fold-changes (infected versus control) on both gene expression platforms. We hypothesized that genes with high differential fold-changes in expression based on RNA-seq analysis should also show high differential fold-changes based on the microarray data. Accordingly, these data would be expected to yield a significant, positive correlation. For this, all 12,294 genes and 5,082 probe sets that passed the filtering criteria across both sample groups for both RNA-seq and microarray analysis, respectively, were considered. Next, we identified all the genes, irrespective of significance, that were common to both data sets based on Ensembl gene ID, this involved matching the Ensembl IDs of the filtered genes in the RNA-seq data set with the Ensembl IDs of the filtered microarray probe sets. In total, 2,265 Ensembl IDs were identified in common between the two platforms. The log_2_ fold-change values for these 2,265 genes (infected versus control) for both the RNA-seq and microarray data, irrespective of the significance of differential expression generated a Spearman correlation coefficient of 0.88 (*P* ≥ 0.001), which underlines the reproducibility and robustness of both platforms for investigations of differential gene expression.

### Comparison of the fold-change and difference in expression values for RNA-seq and microarray data

We next investigated the correlation between fold-change in expression and gene expression levels. This enabled us to determine if the highest fold-changes in gene expression were observed for genes with low levels of expression. For this, we compared the log_2_ expression fold-changes with the log_2_ differences in CPM between infected and control groups for the RNA-seq platform. We also compared the log_2_ expression fold-changes with the log_2_ differences in hybridization intensity between infected and control groups for the microarray platform. We hypothesized that if transcripts with the lowest expression levels gave the highest fold-change values, a negative correlation would be observed between log_2_ expression fold-changes and log_2_ differences for genes displaying increased relative expression in the infected group relative to the control group. Reciprocally, a positive correlation would be expected between log_2_ expression fold-changes and log_2_ differences for genes displaying decreased relative expression for the same group contrast.

For the RNA-seq platform, all 12,294 genes that passed filtering were used. Of these, 6,243 displayed an increase in relative expression and 6,051 displayed a decrease in relative expression, irrespective of significance. Spearman rank correlation coefficients of 0.35 and −0.43 were observed for genes displaying increased and decreased relative expression, respectively (*P* ≤ 0.001). For the microarray platform, we used all 5,082 probe sets that passed filtering of which 2,551 displayed an increase in expression and 2,531 displayed a decrease in expression, irrespective of significance. Spearman rank correlation coefficients of 0.61 and −0.64 were observed for genes displaying increased and decreased relative expression, respectively (*P* ≤ 0.001). The correlation coefficients for both the RNA-seq and microarray platforms for genes with increased and decreased relative expression do not support the hypothesis detailed above; therefore, we conclude that there is no obvious relationship between gene expression level and fold-change in expression.

### Dynamic range of RNA-seq and microarray data

To investigate the dynamic range of the RNA-seq and microarray platforms, the log_2_ reads per kilobase per million (RPKM) from the RNA-seq data and the log_2_ intensities from the microarray data were used; only genes and probe sets that passed the filtering criteria (12,294 and 5,082, respectively) were considered for this analysis. The lowest expression value was subtracted from the highest expression value for each platform. For the RNA-seq platform, the gene displaying the lowest expression level was *MUC5B* (log_2_ RPKM of −6.49), while the gene with the highest value was *COX1* (log_2_ RPKM of 13.46); this yielded a log_2_ dynamic range of 19.96. It is important to note that as RPKM values are proportions, values <1.0 will yield a negative result when log-transformed. For the microarray platform, the probe set with the lowest log_2_ intensity was Bt.29403.1.s1_at (4.89) and the probeset with the highest log_2_ intensity was AFFX-Crex-5_at (13.90), yielding a log_2_ dynamic range of 9.01. Therefore, the dynamic range of RNA-seq for the current study is almost 2,000-fold greater than the microarray.

## Discussion

Whole-genome transcriptional profiling has been successfully used to study human and BTB and has facilitated high-resolution analysis of the host genes and cellular pathways that are activated and perturbed in response to mycobacterial pathogens (11–13, 17, 38–42). The target tissue for studies of the host response has generally been peripheral blood collected from infected and non-infected individuals or animals; peripheral blood provides an easily accessible biological sample that reflects the host immunological and pathological changes induced at the site of infection (9). For example, Berry and colleagues identified a microarray-derived 393 transcript biomarker signature, characterized by type 1 interferon-inducible genes, which discriminated active human TB patients from latently infected and healthy control individuals (38). Furthermore, microarray-based comparative analysis of the peripheral blood transcriptome of active human tuberculosis cases and sarcoidosis patients (an analogous granulomatous disease of the respiratory tract of unknown etiology) also revealed a transcriptional signature that differentiated these two pathologically similar diseases (39). Similarly for BTB, pan-genomic and immuno-specific microarray analysis of peripheral blood has demonstrated that *M. bovis*-infected and non-infected animals can be unambiguously differentiated by disease status. Moreover, downstream analysis of the DE genes provided functional genomics evidence that active BTB is associated with the suppression of host innate immune responses and impairment of T-cell signaling (10, 12).

Notwithstanding the remarkable progress in functional genomics studies of the immunobiology and host–pathogen interactions, microarray studies of the host response to mycobacterial infections are not without limitations. For example, microarrays are limited to analysis of genes/transcripts for which probes can be generated from functionally annotated genome resources. In addition, quantification of gene expression indirectly from hybridization signal intensities constrains the dynamic range for lowly and highly expressed gene transcripts. Also, the measurement of gene expression can be hampered by probes that differ in their hybridization affinities with the target mRNA and by background non-specific hybridization, particularly for lowly expressed genes (15, 43, 44).

In contrast, RNA-seq technologies, which are based on high-throughput sequencing and subsequent counting of all expressed RNA transcripts present in a biological sample, have several advantages for quantifying RNA abundance and unraveling the complexity of the host transcriptome following mycobacterial infection. These include unbiased global gene expression analysis (the entire transcriptome is normally surveyed), detection of allele-specific expression, and cataloging of novel transcripts, RNA classes (e.g., long non-coding RNA transcripts), and splice variants that are rarely quantifiable using microarray technologies (15, 44). Also, RNA-seq analysis is based on digital counts of reads that map to annotated genes within a reference genome, thus offering a more precise and sensitive method to identify and quantify DE genes than analog microarray hybridization intensities. Consequently, for the present study we have compared the peripheral blood transcriptomes of non-infected control animals and animals naturally infected with *M. bovis* (eight samples per group) using RNA-seq and compared these results to parallel data obtained using the pan-genomic Affymetrix^®^ Bovine Genome Array.

### Functional biology of RNA-seq results

A mean of 13.2 million 78-mer reads per individually barcoded RNA-seq library was obtained and this yielded a mean of 1.03 Gb of sequence data per library. A mean of 11.8 million reads (89% of the mean number of library reads) per library mapped to unique locations in the bovine UMD3.1 reference genome. This is higher than previously reported comparable studies: Nalpas and colleagues observed that 63.6% of reads mapped to unique UMD3.1 regions and Churbanov and colleagues observed that 71.2% of reads mapping to the Btau 4.0 reference genome (17, 45). Analysis of the gene coverage from these uniquely mapped reads revealed that 17,792 genes from a total of 24,616 annotated bovine genes gave at least 1 sequence read in at least 1 of the 16 individual libraries. As accurate quantification of gene expression is reliant on sequencing depth, whereby low sequencing coverage can lead to the generation of false-positive DE genes (type I errors) (46, 47), we have used a stringent sequencing-depth filtering criterion to remove lowly expressed genes. This filtering involved the retention of genes displaying more than one CPM in eight or more individual libraries; 12,294 genes (49.9% of annotated *B. taurus* genes) were retained for downstream differential expression analyses. The number of biological replicates (*n* = 8) and mean sequencing depth per library achieved in the present study is sufficient for the accurate quantification and analysis of DE genes with a corresponding reduction in the number of type I errors due to lowly expressed genes (46–48).

Previous work by our group and others has demonstrated that a functional genomics approach can highlight novel aspects of the complex etiology of *M. bovis* infection in cattle. These studies have confirmed the role of T_H_1-type cytokines and chemokines and innate immune receptors (e.g., TLR genes) in mediating the response to *M. bovis* infection. Moreover, these investigations support the hypothesis that the immunoevasive mechanisms used by the pathogen during infection are reflected in the host transcriptome at the peripheral blood level; in particular, the suppression of innate immune signaling, which leads to an inferior adaptive immune response (10, 12).

In the current study, RNA-seq was used to identify a total of 3,250 DE genes in the infected group relative to the control group; of these, 1,579 genes displayed increased relative expression and 1,671 genes showed reduced relative expression. The number of DE genes identified here through RNA-seq analysis exceeds the number of DE gene previously reported by Killick et al. (12) for the same RNA samples (2,757 DE genes; 1,281 and 1,476 genes displaying increased and decreased relative expression, respectively). This finding emphasizes the increased sensitivity of RNA-seq compared to microarrays for studies of differential gene expression (17, 49).

Gene ontology analysis revealed enrichment for genes involved in inflammation and immunity. T_H_1-type cytokines and chemokines, such as *CXCL6* and *IL8*, were among the top-ranking DE genes based on fold-change in expression; additional innate immune genes, such as *CCL4, CXCR4, CXCR7, IL1A, IL8, IL10*, and *TLR4* also shown increased expression in the *M. bovis*-infected group relative to the controls. Several innate immune genes also displayed reduced relative expression including *CXCL10, DEFB10, IL12, IL18*, and *IL27*. These results suggest that although innate immune genes play a role in mediating the host response to *M. bovis* infection, these genes may also serve as targets for immunomodulation by the pathogen to facilitate survival in the host. For example, *IL12, IL18*, and *IL27* encode cytokines that have all been shown to play key roles in initiating and controlling the adaptive immune response to mycobacterial infection (31, 50); suppression of these genes may result in the development of an inferior cellular response to infection leading to disease progression. The increased relative expression of the *IL10* gene, which encodes an immunosuppressive cytokine, may also result in the suppression of host innate immune responses to infection resulting in mycobacterial persistence within the host (51). Collectively, these findings support our previous work, which hypothesized that the suppression of innate immune expression and signaling limits the initiation and maintenance of an appropriate adaptive immune response, contributing to the progression of BTB disease (10, 12).

Further analysis of the DE genes using the IPA Knowledgebase identified additional cellular mechanisms within several of the top-ranking canonical pathways, which may be subject to immunomodulation by the pathogen, including the *Leukocyte Extravasation Signaling* and *Tec Kinase Signaling* pathways. The *Leukocyte Extravasation Signaling* pathway exhibited a decrease in expression for many genes encoding positive modulators of this pathway, including *SPN* (also known as *CD43*), *ITGAL, ITGB2*, and *PECAM1* (52–55). Leukocyte extravasation refers to the transendothelial migration of activated leukocytes from the blood into infected tissue and is vital for immune surveillance and defense (56). This process, which requires the adherence of leukocytes to the endothelial surface of blood vessels followed by transmigration through the endothelial blood vessel cell layer into the infected tissue, is mediated by chemokines and several cell surface proteins and adhesion molecules including selectins and integrins (57). Within the IPA-identified *Leukocyte Extravasation Signaling* pathway, transcriptional suppression was observed for several leukocyte ligands required for endothelial adhesion during extravasation. *SPN, ITGAL*, and *ITGB2* encode the CD43 and the CD11b and CD18 leukocyte cell surface ligands, respectively [the latter of which can complex with different protein partners to form different integrins such as LFA-1 and MAC1 (58)], which are required for leukocyte adhesion to the endothelial cells and subsequent transmigration of the leukocytes into infected tissue (54, 59, 60). *PECAM1* encodes a selectin protein found at intercellular endothelial junctions and is also required for transmigration across these barriers (52, 61). Lower expression of these ligands may result in reduced leukocyte recruitment to the site of infection, leading to an impaired adaptive immune response to contain or eradicate *M. bovis* infection in the host, ultimately leading to disease progression. In addition, these findings lend further support to the hypothesis that the immunoevasion mechanisms used by the pathogen are reflected in the host transcriptome.

Hematological analysis of blood samples taken from the animals analyzed in the current study showed a significant increase in the mean number of lymphocytes (*P* = 0.001) and a significant decrease in the mean number of monocytes (*P* = 0.002) for the infected animals relative to the control group (12). Conversely, no significant differences were observed in the mean number of neutrophils between the two sample groups (*P* ≥ 0.05). It is therefore likely that many of the gene expression changes observed in the current study reflect differences in white blood cell populations between the sample groups.

### Whole-genome expression profiling: RNA-seq versus microarrays

The PBL-extracted RNA analyzed in the present study using RNA-seq had also been previously analyzed by us using the Affymetrix^®^ GeneChip^®^ Bovine Genome Array (12); this enabled a direct technical comparison between the two platforms. RNA-seq analysis detected 2.3-fold (3,250/1,398) more DE genes with Ensembl IDs compared to the microarray platform. The percentage of overlapping DE genes with Ensembl IDs between the two platforms was estimated at 28.2% for RNA-seq (917/3,250) and 65.6% (917/1,398) for the microarray. Similarly, the concordance rate based on IPA-identified canonical pathways for the two platforms was estimated at 73.7% for the microarray and 50.2% for RNA-seq. These results demonstrate that the majority of DE genes detected using the microarray are also detected by RNA-seq. This finding also highlights the greater number of DE genes uniquely identified by RNA-seq compared to microarrays as previously reported by us and others (17, 62, 63).

In the current study, the greater number of DE genes detected using RNA-seq can be largely attributed to the greater dynamic range of RNA-seq, which enables sensitive detection of lowly, but DE genes between the infected and control groups (46, 47). Notably, the concordance rate for the microarray (65.6%) is higher than that previously reported by our group for monocyte-derived macrophages (MDM) infected *in vitro* with *M. bovis* (17) and by (64), who examined the transcriptome of anti-CD3- and anti-CD28-stimulated human CD4^+^ T cells. The increased microarray concordance rate (based on the number of DE genes) observed for the current study compared to these previous studies is likely due to increased sequencing depth and a greater number of biological replicates (46–48).

Interestingly, two DE genes (0.06% of all RNA-seq DE genes and 0.14% of all microarray DE genes) displayed opposite directions of expression on the two platforms. These discordance rates are lower than that previously reported for RNA-seq and microarray analysis of human cancer cell transcriptomes (65) and may be explained by several technical factors including random error, differences in the transcript isoforms detected by both platforms, and the susceptibility of microarray probes to cross-hybridize with non-specific gene transcripts (66, 67).

### Whole-genome transcriptomics: Biomarker development for *M. bovis* infection

Multidimensional scaling analysis using all RNA-seq genes that passed the filtering criteria unambiguously differentiated animals on the basis of their disease status (Figure 1A). This result is also supported by the microarray data generated (12) and re-analyzed here. These findings suggest that genome-wide expression profiling of peripheral blood from *M. bovis*-infected animals can be used to identify transcriptional biomarkers for the detection of infected animals within herds and thereby augment surveillance strategies in countries where BTB control programs have been implemented. In addition, recent work has demonstrated that circulating serum or plasma microRNAs may serve as a complementary source of robust biomarkers for tuberculosis and other infectious diseases (68–72). Notwithstanding this, further work using large PBL sample panels from additional animals infected with *M. bovis* and other microbial pathogens will be required to identify and validate robust *M. bovis*-specific transcriptional signatures of infection.

## Conflict of Interest Statement

The authors declare that the research was conducted in the absence of any commercial or financial relationships that could be construed as a potential conflict of interest.

## Supplementary Material

The Supplementary Material for this article can be found online at http://www.frontiersin.org/Journal/10.3389/fimmu.2014.00396/abstract

## Footnotes

^1^http://www.bioinformatics.babraham.ac.uk/projects/fastqc

^2^http://www.ingenuity.com
