# Supplementary material for: RNA-seq Transcriptional Profiling of Peripheral Blood Leukocytes from Cattle Infected with Mycobacterium bovis
Source: Front Immunol. 2014 Aug 26;5:396. doi: 10.3389/fimmu.2014.00396 (PMC4143615; doi:10.3389/fimmu.2014.00396)
Supplement: Supplementary file 1 [file Data_Sheet1.ZIP › Supplementary Material.pdf]

Supplementary Material

RNA-seq transcriptional profiling of peripheral blood leukocytes from cattle infected with *Mycobacterium bovis*

Kirsten E. McLoughlin[1], Nicolas C. Nalpas[1], Kévin Rue-Albrecht[1], John A. Browne[1], David A. Magee[1], Kate E. Killick[1], Stephen D.E. Park[1,2], Karsten Hokamp[3], Kieran G. Meade[4], Cliona O'Farrelly[5], Eamonn Gormley[6], Stephen V. Gordon[7,8] and David E. MacHugh[1,8]

[1] Animal Genomics Laboratory, UCD School of Agriculture and Food Science, University College Dublin, Belfield, Dublin 4, Ireland.
[2] Current address: IdentiGEN Ltd., Unit 2, Trinity Enterprise Centre, Pearse St, Dublin 2 Ireland.
[3] Smurfit Institute of Genetics, Trinity College, Dublin 2, Ireland.
[4] Animal & Bioscience Research Department, Animal & Grassland Research and Innovation Centre, Teagasc, Grange, Dunsany, County Meath, Ireland.
[5] Comparative Immunology Group, School of Biochemistry and Immunology, Trinity Biosciences Institute, Trinity College, Dublin 2, Ireland.
[6] Tuberculosis Diagnostics and Immunology Research Centre, UCD School of Veterinary Medicine, University College Dublin, Belfield, Dublin 4, Ireland.
[7] UCD School of Veterinary Medicine, University College Dublin, Belfield, Dublin 4, Ireland.
[8] UCD Conway Institute of Biomolecular and Biomedical Research, University College Dublin, Dublin 4, Ireland.

*** Correspondence:** David E. MacHugh, Animal Genomics Laboratory, Veterinary Science Centre, University College Dublin, Dublin 4, Ireland.

david.machugh@ucd.ie

1. Supplementary tables

Table S1: Illumina® RNA-seq library information for 16 *M. bovis*-infected and non-infected bovine PBL samples. [**Table_S1.xlsx**]

Table S2: DE genes detected in *M. bovis*-infected animals compared to the control non-infected group using RNA-seq. Datasheet tab 1 and tab 2 contain genes with increased and decreased relative expression, respectively (ranked according to *P*-value [smallest to largest]). [**Table_S2.xlsx**]

Table S3: Canonical pathways identified using IPA for RNA-seq DE gene results from *M. bovis*-infected and non-infected control animals (statistically significant pathways (adjusted $P \leq 0.05$) shown and ranked according to *P*-value [smallest to largest]). [**Table_S3.xlsx**]

Table S4: DE genes detected in *M. bovis*-infected animals compared to the control non-infected group using the Affymetrix® GeneChip® Bovine Genome Array. Datasheet tab 1 and tab 2 contain genes with increased and decreased relative expression, respectively (ranked according to *P*-value [smallest to largest]). [**Table_S4.xlsx**]

Table S5: Canonical pathways identified using IPA for microarray DE gene results from *M. bovis*-infected and non-infected control animals (statistically significant pathways (adjusted $P \leq 0.05$) shown and ranked according to P-value [smallest to largest]) [**Table_S5.xlsx**]
